# Integration of modern genetic knowledge and technology into public health in India

**DOI:** 10.4103/0971-6866.69325

**Published:** 2010

**Authors:** Kanjaksha Ghosh, Ajit Gorakshakar

**Affiliations:** National Institute of Immunohematology (ICMR), 13^th^ Floor, New Multistoryed Building, K.E.M Hospital Campus, Parel, Mumbai, India

Public health or community medicine is one of the triumphs of modern medicine.[1] Major control and eradication of many diseases from the face of the earth, using the principles of public health, that is, personal hygiene, water hygiene, milk hygiene, nutritional activities with staple food items (iodization of salt, Folate fortification of cereals, etc.), antenatal care, school health programs, environmental sanitation, immunization, basic principles of investigation, and containment of epidemics have given us rich dividends, and as we control common infectious diseases and nutritional deficits or excesses increasingly, we are left with disorders and disabilities that are largely of genetic origin, barring road traffic and other accidents, which also take a substantial toll of human life.

During the last 30 years there have been unprecedented developments in the understanding of how genes function. How the function of one gene affects the function of a single or many other genes (epistasis), how the risk of multigenic inheritance can be calibrated, and how the science of genomics gives us the activity of the whole genome and various mutations, while *single-nucleotide polymorphisms* (SNP’s) allow risk stratification and prognostication in various monogenic and mutligenic disorders.

Furthermore, various gene-based technologies have been so simplified that today any modern healthcare facility is capable of using them.

After the human genome project was completed there was a flurry of activities to search for genes for various disorders, and many disorders that were considered to be contributed by one gene were found to be heterogenous in nature, involving different genes from different families (Hemochromatosis). Even a monogenic disorder was found to be heterogenous when the gene pathology was considered (More than 200 mutations were described for β thalassemia alone).[2]

Now the major challenge to the Government of any country and the other caregivers is to use this humongous amount of genetic data for the benefit of mankind, for improving the personal and public healthcare system. There is a need to give a deep thought to how this can be done?

Many serious genetic disorders need huge financial outlays to keep the patient alive, prenatal diagnosis is available as a preventive measure.[3,4] For those who cannot undergo prenatal diagnosis, because of various reasons, preconceptional diagnosis is possible.[5] Many genetic disorders can now be detected in the presymptomatic stage, hence, preventive measures can be undertaken. Multidrug-resistant TB is threatening the health infrastructure in many countries and this strain of TB bacillus can be easily diagnosed using modern molecular biology techniques; hence, this kind of a laboratory is a public health necessity. Pharmaceutical industries are churning out various new chemical entities, the side effects of such drugs or their activities depend on the genetic background of the patients. For quite a few drugs a gene-based analysis is now available.[6] Community participation in curing severe diseases is already visible in hemotopoietic stem cell transplantation, where millions of unrelated donors are available through various registries, and matching of the recipients and donors is done through molecular biological techniques.

Previously genetic diseases were thought to be incurable and proper genetic counseling was the only recourse. However, nowadays, many intelligent therapies have come up, such as, enzyme replacement therapy and substrate reduction therapy for Gaucher’s[7] disease. Epigenetic silencing or activation of certain genes as a possible approach is also being explored. Hydroxyurea therapy in sickle cell anemia is one such example.[8]

In addition to the all that has been stated earlier, there is a huge area of negative impact with regard to the gene-based practice of medicine, for example, discrimination, resource allocation, commodification, eugenics, and genetic policemanship are some of the concerns.[9] In this issue of the Journal, Ashwini has reviewed the existing literature on the utilization of genetic and genomic medicine for the welfare of the general public, and also how it can be integrated into the current healthcare system.[10]

In India, the situation is different. Earlier, genetics in public health was related to screening for some genetic disorder like sickle cell anemia or thalassemia. However, there are several other diseases such as Tay-sach’s disease, mental retardation, and cystic fibrosis, where the proper prevalence is not known. Once it is found one can think about the management and prevention through prenatal diagnosis.

Research in genetics and genomics has opened up several molecular genetic tests, which have entered our healthcare system. However, in a country like India, more than 75% of the people live in rural areas and do not get the benefit of this advanced testing. In fact their needs are different. In ‘high-risk’ communities for β-thalassemia or in tribal pockets with a high prevalence of sickle hemoglobin, the main requirements are, creating an awareness of the disorders and genetic counseling, which can help to prevent the transmission of these disorders to the next generation.[11]

The Indian Institute of Public Health, which is an autonomously governed body with branches at Hyderabad, Delhi, Shillong, and so on, are mainly running campaigns against chewing tobacco and conducting operational research on TB, HIV, and other non-communicable diseases. They are also conducting courses for biostatisticians and epidemiologists, however, not much attention is given to genetics. Educating patients, parents, voluntary organizations, as well as the general public about various genetic disorders, and their management and prevention, is an important step in creating awareness. Efforts should be targeted toward identifying the affected families and their needs, and then offering them proper counseling. These are the preliminary goals of integrating genetics into the public health in India. Once they are achieved one can think of genomic medicine and other advanced studies to detect genetic disorders, novel genetic association, and the like, at the population level.
